# Low Energy Electron Irradiation Is a Potent Alternative to Gamma Irradiation for the Inactivation of (CAR-)NK-92 Cells in ATMP Manufacturing

**DOI:** 10.3389/fimmu.2021.684052

**Published:** 2021-06-04

**Authors:** Lia Walcher, Ann-Kathrin Kistenmacher, Charline Sommer, Sebastian Böhlen, Christina Ziemann, Susann Dehmel, Armin Braun, Uta Sandy Tretbar, Stephan Klöß, Axel Schambach, Michael Morgan, Dennis Löffler, Christoph Kämpf, Conny Blumert, Kristin Reiche, Jana Beckmann, Ulla König, Bastian Standfest, Martin Thoma, Gustavo R. Makert, Sebastian Ulbert, Uta Kossatz-Böhlert, Ulrike Köhl, Anna Dünkel, Stephan Fricke

**Affiliations:** ^1^ Department for GMP Process Development/ATMP Design, Fraunhofer Institute for Cell Therapy and Immunology (IZI), Leipzig, Germany; ^2^ Fraunhofer Institute for Toxicology and Experimental Medicine (ITEM), Department for Preclinical Pharmacology and Toxicology, Member of the German Center for Lung Research (DZL), Biomedical Research in Endstage and Obstructive Lung Disease (BREATH) research network, Hannover, Germany; ^3^ Institute of Cellular Therapeutics, Hannover Medical School, Hannover, Germany; ^4^ Institute of Experimental Hematology, Hannover Medical School, Hannover, Germany; ^5^ Department for Diagnostics, Fraunhofer Institute for Cell Therapy and Immunology (IZI), Leipzig, Germany; ^6^ Institute for Clinical Immunology, University of Leipzig, Leipzig, Germany; ^7^ Division for Medical and Biotechnological Applications, Fraunhofer Institute for Organic Electronics, Electron Beam and Plasma Technology (FEP), Dresden, Germany; ^8^ Department for Laboratory Automation and Biomanufacturing Engineering, Fraunhofer Institute for Manufacturing Engineering and Automation (IPA), Stuttgart, Germany; ^9^ Department for Vaccines and Infection Models, Fraunhofer Institute for Cell Therapy and Immunology (IZI), Leipzig, Germany; ^10^ Fraunhofer Institute for Cell Therapy and Immunology (IZI), Leipzig, Germany

**Keywords:** NK-92, CAR-NK-92, low energy electron irradiation, gamma irradiation, acute myeloid leukemia, chimeric antigen receptor, immune cell therapy, off-the-shelf therapy

## Abstract

**Background:**

With increasing clinical use of NK-92 cells and their CAR-modified derivatives in cancer immunotherapy, there is a growing demand for efficient production processes of these “off-the-shelf” therapeutics. In order to ensure safety and prevent the occurrence of secondary tumors, (CAR-)NK-92 cell proliferation has to be inactivated before transfusion. This is commonly achieved by gamma irradiation. Recently, we showed proof of concept that low energy electron irradiation (LEEI) is a new method for NK-92 inactivation. LEEI has several advantages over gamma irradiation, including a faster reaction time, a more reproducible dose rate and much less requirements on radiation shielding. Here, LEEI was further evaluated as a promising alternative to gamma irradiation yielding cells with highly maintained cytotoxic effector function.

**Methods:**

Effectiveness and efficiency of LEEI and gamma irradiation were analyzed using NK-92 and CD123-directed CAR-NK-92 cells. LEE-irradiated cells were extensively characterized and compared to gamma-irradiated cells *via* flow cytometry, cytotoxicity assays, and comet assays, amongst others.

**Results:**

Our results show that both irradiation methods caused a progressive decrease in cell viability and are, therefore, suitable for inhibition of cell proliferation. Notably, the NK-mediated specific lysis of tumor cells was maintained at stable levels for three days post-irradiation, with a trend towards higher activities after LEEI treatment as compared to gamma irradiation. Both gamma irradiation as well as LEEI led to substantial DNA damage and an accumulation of irradiated cells in the G2/M cell cycle phases. In addition, transcriptomic analysis of irradiated cells revealed approximately 12-fold more differentially expressed genes two hours after gamma irradiation, compared to LEEI. Analysis of surface molecules revealed an irradiation-induced decrease in surface expression of CD56, but no changes in the levels of the activating receptors NKp46, NKG2D, or NKp30.

**Conclusions:**

The presented data show that LEEI inactivates (CAR-)NK-92 cells as efficiently as gamma irradiation, but with less impact on the overall gene expression. Due to logistic advantages, LEEI might provide a superior alternative for the manufacture of (CAR-)NK-92 cells for clinical application.

## Introduction

The natural killer cell line NK-92 is derived from the peripheral blood of a lymphoma patient and is used as an anti-cancer advanced therapy medicinal product (ATMP) due to its high cytotoxic activity against tumor cells and its “off-the-shelf” availability (1). Further enhancement of NK-92 efficacy was achieved by chimeric antigen receptor (CAR)-modification of the cell line (2). Currently, CAR-NK-92 cells are investigated as alternative effector cells in comparison to autologous CAR-T cells. For acute myeloid leukemia (AML), a possible target is the interleukin 3 receptor alpha (IL-3Rα, also known as CD123), which is overexpressed in AML [summarized in (3)] and expression levels were shown to correlate with clinical prognosis and outcome (4–6). Previously, we demonstrated the *in vitro* efficacy of CD123-directed CAR-NK-92 cells (7).

Due to the malignant origin of NK-92 cells, cell inactivation prior to their application is indispensable in order to stop cell proliferation and prevent possible tumorigenesis (2, 8, 9). For all clinical trials published so far, the gold standard has been gamma irradiation of NK-92 cells at a dose of 10 Gy prior to infusion (10, 11). Physically, the effect of ionizing radiation (IR) is based on breakage of atomic bonds with subsequent formation of radicals. The damaging effects are therefore categorized into primary reactions, i.e. direct DNA damage, and secondary reactions from water radiolysis products, including reactive oxygen species (ROS) (12, 13). Induced structural DNA damage thereby includes single- or double-strand breaks (DSBs), cross-linkage breaks and nucleotide degradation (13–15). As a result, apoptosis, necrosis and senescence (13, 16) as well as a strong impairment of cell membranes and proteins can occur (13, 17).

In addition to the conventional gamma irradiation, low energy electron irradiation (LEEI) has emerged as a novel method for NK-92 cell inactivation (18). Since the penetration depth of the accelerated low energy electrons is limited to < 1 mm in water (19), a thin film of liquid has to be generated to ensure complete irradiation (18). Compared to gamma irradiation, LEEI allows delivery of a high dose rate, which leads to a shorter treatment time (18, 20). One of the greatest benefits of LEEI is the generation of only small amounts of secondary radiation (the Bremsstrahlung, X-rays) (18). This is the reason why LEEI facilities do not need to be equipped with complex shielding systems and therefore can easily be implemented in basic laboratories, even as an in-line tool in GMP environments (21). Furthermore, the applied LEEI doses can be adjusted by current intensity, resulting in a well-controllable on-off-process with a very good reproducibility, a great advantage over gamma radiation, which is caused by the spontaneous decay of radioactive material (18).

Many preclinical and all clinical studies involving NK-92 cells employ IR as a tool for cell inactivation, but few reports are available on cellular effects caused by this treatment. In our present study, an extensive characterization of both gamma- and LEE-irradiated NK-92 as well as CD123-directed CAR-NK-92 cells with regard to descriptive and functional attributes was performed. Our data indicate the potential of LEEI for manufacturing enhanced NK-92-based cellular therapeutics with preserved cytotoxic activity.

## Materials and Methods

### Cell Culture of Wild Type and Redirected Cells

NK-92, K562, and KG-1 cell lines were purchased from the German Collection of Microorganisms and Cell Cultures (DSMZ). CD123-directed CAR-NK-92 were generated via alpharetroviral transduction (7) and sorted thereafter (22). K562 and KG-1 cells were cultured in RPMI 1640 medium (Thermo Fisher Scientific), supplemented with 10% heat-inactivated fetal bovine serum (FBS, Sigma-Aldrich). NK-92 and CD123-directed NK-92 cells were cultured in X-VIVO™ 10 medium with recombinant transferrin and L-glutamine, without gentamicin and phenol red, xeno-free, (Biozym Scientific GmbH), supplemented with 5% heat-inactivated human serum (Sigma-Aldrich) and 100 IU/mL IL-2 (Proleukin S, Novartis). All cell lines were maintained at 37°C and 5% CO_2_ and were regularly checked for mycoplasma contamination.

### Cell Irradiation

For LEEI, cells were irradiated as described previously (18), using a custom-built irradiation device, equipped with an electron emitter (type EBA 300/270/4, ebeam Technologies, Switzerland) and a custom-made bag module. Cell suspensions were placed into sterile disposable bags and processed at a bag transportation velocity of 5 mm/s through the LEEI-area. Irradiation parameters (200 keV and 0.01 mA, 0.03 mA, or 0.05 mA) correspond to doses of 2.2 Gy, 6.6 Gy, or 11 Gy, respectively, which were calculated based on dosimetry at higher doses and a reduction factor of 0.01 for the irradiation with a slit diaphragm, as described previously (18).

Gamma irradiation was performed at a dose of 10 Gy, using a Cs-137 source (GSR C1, serial number 09/13, Gamma Service Medical GmbH), since 10 Gy represents the clinical gold standard. For both LEEI and gamma irradiation, up to 1.5 × 10^8^ cells were irradiated in a volume of 20 mL of cell culture medium. Directly after irradiation, the cell concentration was adjusted to 0.3 × 10^6^ viable cells/mL with fresh cell culture medium. Afterwards, the cell concentration was adjusted 2 - 3 times per week to 0.3 – 0.5 × 10^6^ viable cells/mL. In order to check for irradiation-independent effects, control cells were processed in parallel without applying irradiation (process control, data not shown).

### Cell Number and Viability

In order to monitor cell inactivation after irradiation, cell counts and viability were determined using a Countess II Automated Cell Counter (ThermoFisher Scientific). According to the manufacturer’s instructions, cells were stained with trypan blue solution (Thermo Fisher Scientific) at a ratio of 1:2. All counts were performed in technical triplicates. Alternatively, as indicated in the figure legend, a CASY cell counter & analyzer (OMNI Life Science) was used according to the manufacturer’s instructions.

### Flow Cytometry Analysis

For flow cytometric analysis of cell surface markers or cell death, the following antibodies were used: APC-Cy7 anti-human NKG2D (BioLegend), PE anti-human NKp30, APC anti-human NKp46, BV421 anti-human CD56, APC Annexin V and 7-aminoactinomycin D (7-AAD) (all from BD Bioscience). Up to 5 × 10^5^ cells were washed with 1 × phosphate-buffered saline (PBS) supplemented with 5% FBS and 2.5 mM ethylenediaminetetraacetic acid (EDTA). Antibody staining was performed at 1:15 – 1:100 dilution for 30 min at room temperature, protected from light, followed by two further washing steps. For Annexin V staining, cells were resuspended in 1 × Annexin V binding buffer (BD Bioscience), Annexin V was added for 20 min, and cells were washed again in Annexin V binding buffer. Ten minutes prior to measurement, 7-AAD was added.

Data acquisition was performed using a BD FACSCanto™II and data were analyzed using BD FACSDiva™ software. CS&T beads (BD Bioscience) were used for quality control. Recorded events were gated for living cells (FSC-A × SSC-A), then for single cells (FSC-H × FSC-A), and finally for the respective marker. At least 10,000 events were recorded in the live gate. For determination of irradiation-induced cell death, cells were gated from all events (FSC-A × SSC-A) excluding debris.

### Cytotoxicity Assay

Cellular cytotoxicity was determined by chromium release assay. Target cells were incubated with 50 µCi of chromium-51 radionuclide (Hartmann analytic) per 6 × 10^5^ cells for 2 h at 37°C and 5% CO_2_. Cells were washed three times and coincubated with effector cells at an effector to target (E:T)-ratio of 5:1 for 4 h at 37°C and 5% CO_2_. To determine spontaneous chromium-51 release, target cells were incubated with medium; to determine maximum release, cells were incubated with 1% Triton-X100. After coincubation, cells were centrifuged and 50 µL of supernatant were added to 150 µL of scintillation mix (Optiphase HiSafe, Perkin Elmer). Scintillation counts were measured for 1 min/well using a Perkin Elmer MicroBeta Trilux 1450 LSC & Luminescence Counter. Specific lysis was calculated using the following formula: Specific lysis = [(test release – spontaneous release)/(maximum release – spontaneous release)] × 100.

### Multiplex Bead Assay for Quantification of Soluble Analytes

Released levels of soluble analytes from NK-92 cells and CD123-directed CAR-NK-92 cells were quantified by the bead-based LEGENDplex™ human CD8/NK panel immunoassay (BioLegend). Effector and target cells were coincubated at a ratio of 5:1. To determine basal level of analytes, effector and target cells were cultured separately. After 2 h of incubation at 37°C, the plate was centrifuged and 100 µL supernatant were collected. To avoid contaminations, the supernatant was centrifuged again and final supernatant was removed and stored at -80°C. Afterwards, the assay was performed according to the manufacturer’s instructions. For qualification and quantification of collected data, analysis was performed with the LEGENDplex™ Data Analysis Software.

### Analysis of Metabolism

For analysis of metabolic capacity, the colorimetric WST-1 assay (Roche) and the luminescence-based CellTiter-Glo^®^ assay (Promega) were performed according to the manufacturer’s instructions. Plates were incubated at 37°C for 3.5 h and were measured directly by a plate reader (Tecan Infinite M200).

### Alkaline Comet Assay

For further assessment of DNA-DSBs, alkaline comet assays were performed 2 h and 24 h after irradiation under red light to avoid UV-induced unspecific DNA damage according to Ziemann et al. (23). Per sample, 150,000 cells were collected and resuspended in 80 µL of 0.75% (w/v) pre-conditioned low-melting-point agarose (LMA, Sigma-Aldrich), and applied to slides pre-coated with normal-melting-point agarose (NMA, Sigma-Aldrich). These gels were covered with coverslips, allowed to set at 4°C, followed by addition of a second LMA layer and another solidification step. The coverslips were removed and the slides were incubated in lysis solution overnight at 4°C. After three washing steps in electrophoresis buffer (pH > 13), slides were placed in a pre-cooled electrophoresis chamber, and DNA was allowed to unwind for 20 min before electrophoresis was carried out at 26 V and 300 mA for 20 min. Finally, slides were neutralized and stained with ethidium bromide solution (20 µg/mL, Merck-Millipore). Slides were semi-automatically analyzed for occurrence of DNA damage using an Axioskop fluorescence microscope (Carl Zeiss) and the Comet Assay III software (Perceptive Instruments). Tail intensity (TI) of 100 nuclei per slide was determined, precluding so-called ‘hedgehogs’ or overlapping nuclei/comets from analysis.

### Propidium Iodide (PI) Staining

For assessment of cell cycle phase distribution, up to 5 × 10^5^ cells were fixed at the indicated time point after irradiation using 70% ethanol. After two washing steps with 0.1% bovine serum albumin in PBS, RNA was digested with RNase A (100 µg/mL final concentration, Thermo Fisher Scientific) and PI was added at a final concentration of 50 µg/mL.

### Light Microscopy

Microscopic images were recorded on a Leica DMIL inverse microscope with a Leica EC3 camera and analyzed using Leica LAS EZ software.

### Next Generation Sequencing (NGS)

For RNA sequencing, 10^6^ cells per sample were collected 2 h post-treatment. Frozen cell pellets (-80°C) were resuspended in Qiazol and RNA was extracted according to the miRNeasy mini protocol (Qiagen). After two steps of DNase-digestion (TURBO DNA free Kit, Ambion), extracted RNA was quantified using a Qubit RNA-Kit and the DeNovix instrument (Biozym). RNA quality was analyzed on a Bioanalyzer 2100 instrument (Agilent Technologies). For subsequent RNA-sequencing analyses, 500 ng of total RNA per sample were used. Library preparation was conducted using Truseq-Stranded mRNA Sample Prep kit (Illumina, Inc, San Diego, CA) according to the manufacturers’ protocol. Molarity of each library was calculated and equal amounts were pooled and used for sequencing (10 pM). Sequencing was performed with 2 × 101-bp paired-end reads using Rapid SBS v2 chemistry on a HiSeq 2500 (Illumina). One Rapid flowcell à 2 lanes was sequenced with 18 pooled libraries. Reads were demultiplexed by Illumina’s bcl2fastq (v2.19.0.316). Adapter sequences were removed from reads by adapterremoval (v2.3.0 using parameters –trimns, –trimqualities, –minquality ‘20’, and –minlength ‘30’) (24). HISAT2 (v2.1.0) with parameters –fr and rna-strandness: RF was used to align reads against the human genome hg38 (GENCODE release 31) (25, 26). Number of reads per gene were counted by htseq-count (v0.11.2) using parameters –mode intersection-strict, –stranded yes and –type exon (27). These steps were orchestrated by the workflow-manager uap (28). Differential gene expression analysis was performed using DESeq2 (v1.30.1) (29). Raw read counts were normalized and variance stabilized. False discovery rate (FDR) was controlled by Benjamini-Hochberg adjustment.

Over-representation analyses (ORA) was performed to identify signalling pathways enriched with genes significantly differentially regulated (FDR < 0.01) between LEEI or gamma irradiation *vs*. control samples. For assignment of genes to pathways the curated database Reactome pathway database (30) was used. Over-representation analyses was conducted using the R-packages ReactomePA (v1.34.0) (31) and clusterProfiler (v3.18.1) (32). Pathways with less than 10 or more than 500 genes were excluded. All pathways with an adjusted p-value < 0.01 for the hypergeometric test were reported as enriched pathways.

### Statistical Analysis

GraphPad Prism version 6 or 8 (GraphPad Software, Inc., USA) was used for statistical analysis of all data. GraphPad QuickCalcs was used for determination of outliers with a significance level of α = 0.05. Normal distribution was determined using D’Agostino and Pearson omnibus normality test. In case of normally distributed data, analysis of variance (ANOVA) was used; in case of not normally distributed data, Kruskal-Wallis test or Mann-Whitney test was used, as indicated. P-values were adjusted for multiple comparisons by Dunn’s multiple comparisons test and are symbolized by asterisks as follows: * for p ≤ 0.05, ** for p ≤ 0.01, *** for p ≤ 0.001, and **** for p ≤ 0.0001.

## Results

### NK-92 and CD123-Directed CAR-NK-92 Cell Proliferation Is Fully Inhibited by LEEI and Gamma Irradiation

**Figure 1:**
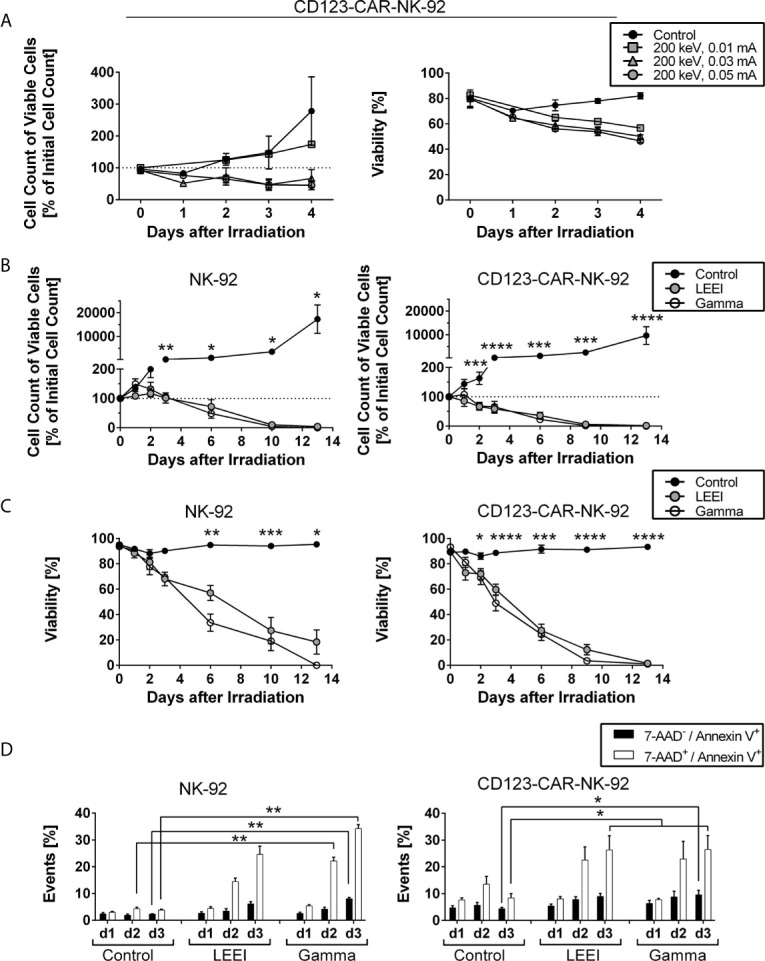
Cell proliferation of NK-92 and CD123-directed CAR-NK-92 is fully inhibited by gamma irradiation and LEEI.** **(A)** Cell count of viable CD123-CAR-NK-92 cells (left) and viability (right) were determined for 4 days for non-irradiated cells (black circles, n = 3) and LEE-irradiated cells at 200 keV and 0.01 mA (grey squares, n = 1), 0.03 mA (grey triangles, n = 3) or 0.05 mA (grey circles, n = 3) on an automated cell counter (CASY). **(B)** Cell count of viable NK-92 (left, n = 5) and CD123-CAR-NK-92 cells (right, n = 9) was determined for 13 days after irradiation for non-irradiated cells (black), LEE-irradiated cells (grey) and gamma-irradiated cells (white) on an automated cell counter (Countess II). **(C)** Viability was measured by trypan blue staining on an automated cell counter (Countess II) for 13 days after irradiation of NK-92 (left, n = 5) and CD123-CAR-NK-92 cells (right, n = 9). Non-irradiated cells (black) were compared to LEE-irradiated cells (grey) and gamma-irradiated cells (white). **(D)** Flow cytometry analysis of LEE- or gamma-irradiated NK-92 (left, n = 4) and CD123-directed CAR-NK-92 (right, n = 7) cells after 7-AAD and Annexin V staining. 7-AAD^-^/Annexin V^+^ (black) and 7-AAD^+^/Annexin V^+^ (white) subpopulations are shown. All values are indicated as means ± SEM, statistical significance is symbolized by asterisks (* for p ≤ 0.05, ** for p ≤ 0.01, *** for p ≤ 0.001, and **** for p ≤ 0.0001, ANOVA or Kruskal-Wallis test adjusted for multiple comparisons by Dunn’s test).

In our previous proof of concept work, it was shown that LEEI is suitable for growth inhibition of NK-92 cells at a dose of 11 Gy (calculated dose for the process parameters 200 keV and 0.05 mA) (18). We here aimed to determine the lowest dose parameters able to efficiently block proliferation of CD123-directed CAR-NK-92 cells by applying LEEI at 200 keV and 0.01 mA, 0.03 mA, or 0.05 mA and analyzing the cell number and viability for 4 days after irradiation (**Figure 1A**). Cells irradiated at an amperage of 0.01 mA showed reduced proliferation and viability, compared to non-irradiated cells, however, inhibition was only partial as cell numbers increased over time. In contrast, cells irradiated at an amperage of 0.03 mA and 0.05 mA showed a decrease in cell number and viability. Based on these results, an amperage of 0.05 mA was used for further experiments, as this dose showed consistent inhibition of cell growth and gave a safety margin to the parameter of 0.01 mA, which is important for implementing LEE irradiation into perspective clinical studies and uses.

To compare LEEI with gold-standard gamma irradiation, NK-92 (n = 5) and CD123-directed CAR-NK-92 cells (n = 9) were irradiated with LEEI or gamma rays using the lowest proliferation-inhibiting doses (11 Gy (calculated dose at 200 keV, 0.05 mA) for LEEI and 10 Gy for gamma irradiation). Cell proliferation (**Figure 1B**) and viability (**Figure 1C**) were analyzed for 13 days. In contrast to non-irradiated control cells, which showed expected cell growth and stable viability over 13 days (cell number > 9,000% of initial cell count on day 13 with a viability of > 90%), irradiated NK-92 and CD123-directed CAR-NK-92 cells showed a significant decrease in proliferation and viability. At day 13 post-irradiation, the number of remaining viable cells relative to the initial cell number approached zero (0.0 ± 0.0% for gamma-irradiated NK-92 cells; 0.2 ± 0.2% for gamma-irradiated CD123-directed CAR-NK-92 cells; 4.2 ± 4.2% for LEE-irradiated NK-92 cells; 1.7 ± 1.3% for LEE-irradiated CD123-directed CAR-NK-92 cells, **Figure 1B**). Correspondingly, the viability decreased significantly until day 13 (0.0 ± 0.0% for gamma-irradiated NK-92, 1.0 ± 0.8% for gamma-irradiated CD123-directed CAR-NK-92, 18.3 ± 9.6% for LEE-irradiated NK-92 and 1.5 ± 1.0% for LEE-irradiated CD123-directed CAR-NK-92 cells, **Figure 1C**). When cell viability was higher than 0% on day 13, monitoring of cell growth was pursued to ensure complete proliferation inhibition (data not shown). There was a trend towards better maintenance of viable cells of LEEI over gamma irradiation between day 6 and 10 (12.3 – 57.0% and 3.6 – 33.6% viability, respectively), but the difference was statistically insignificant.

In order to confirm the decrease in viability observed by trypan blue staining, cell death was analyzed by 7-AAD and Annexin V staining (**Figure 1D**, representative flow cytometry plots shown in **Supplemental Figure S1**). Irradiated NK-92 and CD123-directed CAR-NK-92 cells showed a significant increase in double positive (7-AAD^+^/Annexin V^+^) cells compared to non-irradiated control cells over time. For NK-92 cells (**Figure 1D**, left) and CD123-CAR-NK-92 cells (**Figure 1D**, right), the amount of double positive cells on day 3 increased after LEEI (24.6 ± 3.1%, p = 0.1551 for NK-92; 26.3 ± 5.3%, p = 0.0116 for CAR-NK-92; Kruskal-Wallis test compared to non-irradiated cells) and after gamma irradiation (34.3 ± 1.5%, p = 0.0065 for NK-92; 26.5 ± 5.2%, p = 0.0116 for CAR-NK-92; Kruskal-Wallis test compared to non-irradiated cells), as compared to control cells (3.8 ± 0.4% for NK-92; 8.4 ± 1.6% for CAR-NK-92).

### LEE-Irradiated Cells Show High *In Vitro* Functionality

**Figure 2:**
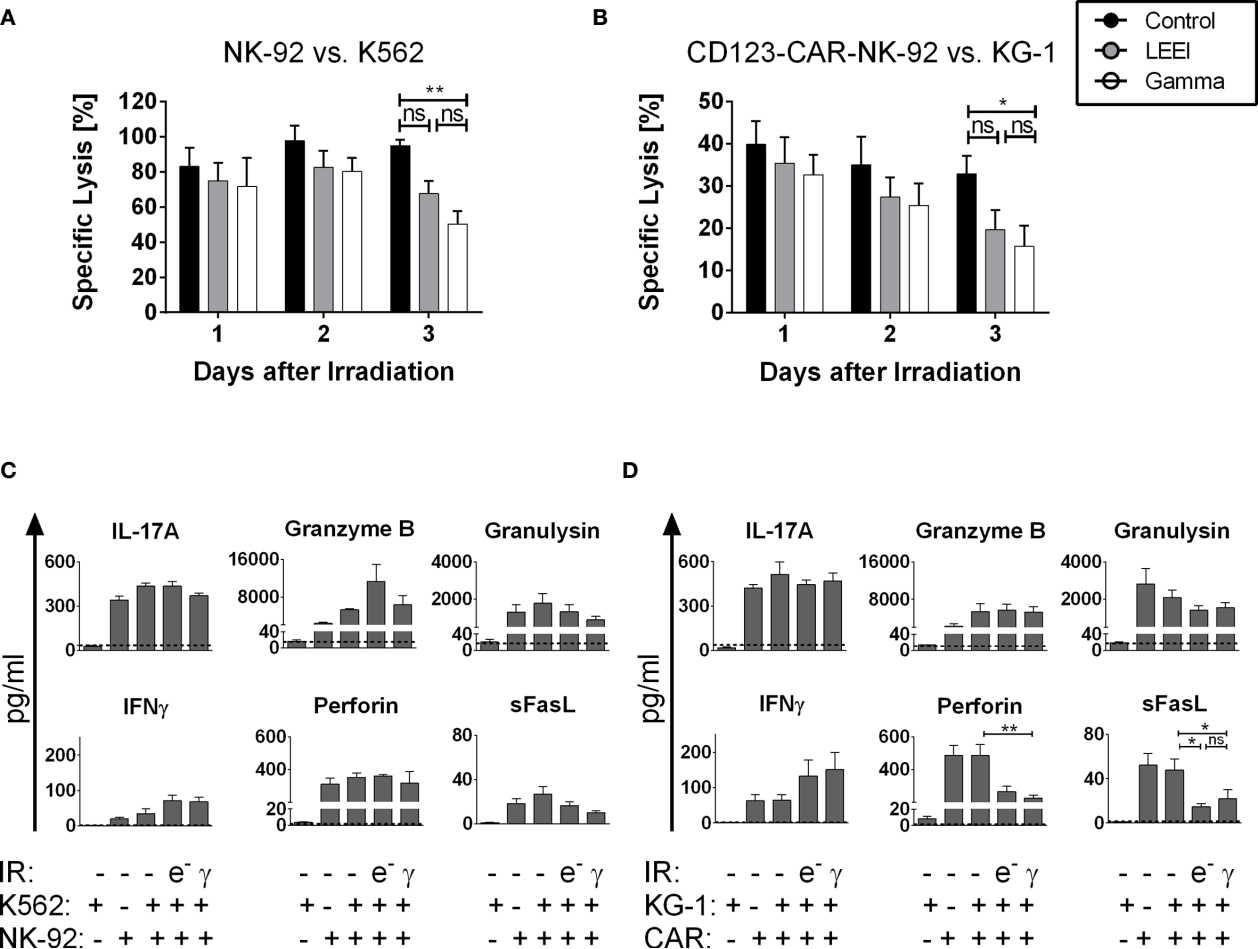
LEE-irradiated cells show high *in vitro* functionality. (A+B): Cytotoxicity was measured *via* specific lysis of K562 cells by NK-92 cells (**A**, n = 5) or specific lysis of KG-1 cells by CD123-CAR-NK-92 cells (**B**, n = 9) by chromium-release-assay for 3 days after LEEI (grey) or gamma irradiation (white). Non-irradiated cells (black) were used as a control. Cells were coincubated at an effector to target (E:T)-ratio of 5:1. (C+D): Three days after LEEI or gamma irradiation, NK-92 (**C**, n = 4) or CD123-CAR-NK-92 (**D**, n = 7) cells were cocultivated with the target cell line K562 or KG-1 in an E:T ratio of 5:1 for 2 h, respectively. As controls, non-irradiated NK-92 or CD123-CAR-NK-92 cells with and without specific target cell stimulation, and K562 and KG-1 cells alone were used. Supernatant was harvested and analyzed with a LEGENDplex human CD8/NK panel. Dotted line represents detection limit of this assay. Values are indicated as means ± SEM, statistical significance is symbolized by asterisks (ns for p > 0.05, * for p ≤ 0.05, ** for p ≤ 0.01, Kruskal-Wallis test adjusted for multiple comparisons by Dunn’s test).

A crucial necessity for the therapeutic efficacy of NK cells is the maintenance of the cytotoxic potential. Based on the application regimens used in clinical trials of NK-92 and CAR-NK-92 cells, we defined a therapeutic window of up to three days post-irradiation. Therefore, cytotoxic activity mediated by NK-92 cells against K562 target cells was determined on days 1, 2, and 3 after gamma irradiation or LEEI by chromium release assays (**Figure 2A**). For the first two days after irradiation, the cytotoxic activity of the NK-92 cells against K562 cells was not significantly impaired (specific lysis = 97.7 ± 8.7% for non-irradiated cells, 82.6 ± 9.6% for LEE-irradiated cells and 80.2 ± 7.9% for gamma-irradiated cells on day 2). Three days after irradiation, however, a significant reduction of cell-mediated cytotoxicity was detected in gamma-irradiated NK-92 cells (50.3 ± 7.5%, p = 0.0098 compared to non-irradiated cells, Kruskal-Wallis test). In contrast, LEE-irradiated cells showed a preserved cytotoxic activity on day 3 compared to the non-irradiated control (94.9 ± 3.5% for non-irradiated cells, 67.8 ± 7.0% for LEE-irradiated cells, p = 0.2327, Kruskal-Wallis test). For CD123-directed CAR-NK-92 cells, cytotoxicity against the CD123-expressing AML cell line KG-1 was measured for 3 days post-irradiation (**Figure 2B**). As for NK-92 cells, no significant decrease in cytotoxic activity was observed during the first 2 days post-irradiation, compared to non-irradiated cells (specific lysis = 35.0 ± 6.7% for non-irradiated cells, 27.4 ± 4.7% for LEE-irradiated cells and 25.4 ± 5.1% for gamma-irradiated cells on day 2). On day 3, cytotoxic activities decreased to 15.7 ± 4.9% for gamma-irradiated cells (compared to 32.9 ± 4.3% for non-irradiated cells, p = 0.0454, ANOVA), and to 19.7 ± 4.6% for LEE-irradiated cells (p = 0.1080 compared to non-irradiated cells, ANOVA). Beyond the therapeutic window of three days post-irradiation, residual cytotoxic activity was detected six days post-irradiation in NK-92 cells (37.50 ± 10.34% for LEE-irradiated cells and 40.15 ± 7.79% for gamma-irradiated cells, n = 3) and in CD123-directed CAR-NK-92 cells (7.57 ± 6.13% for LEE-irradiated cells and 10.34 ± 5.06% for gamma-irradiated cells, n = 4), (data not shown).

Since cytotoxicity of proliferation-inhibited NK-92 and CD123-CAR-NK-92 cells was reduced on day 3 post-irradiation, it was investigated whether this was caused by altered concentration levels of effector molecules. Cytokine levels were measured by a multiplex bead-based immunoassay 3 days post-irradiation by coculturing NK-92 or CD123-directed CAR-NK-92 cells with K562 or KG-1 cells, respectively (**Figures 2C, D**). In principle, secretion levels of effector molecules, including IL-17A, granzyme B, and granulysin were stable after LEEI and gamma irradiation. In the case of interferon γ (IFNγ), there was even a slight irradiation-induced increase in both cell lines [NK-92 cells and (CD123-directed CAR-NK-92 cells): 34.3 ± 14.2 pg/mL (64.3 ± 15.7 pg/mL) for non-irradiated cells; 71.2 ± 15.8 pg/mL (132.2 ± 46.1 pg/mL) for LEE-irradiated cells; 67.5 ± 13.8 pg/mL (150.9 ± 49.1 pg/mL) for gamma-irradiated cells]. Furthermore, irradiated NK-92 cells secreted constant levels of Perforin and soluble Fas ligand (sFasL), whereas secretion levels of both effector molecules decreased in CD123-directed CAR-NK-92 cells after irradiation [Perforin (sFasL): 485.8 ± 67.6 pg/mL (47.9 ± 10.0 pg/mL) for non-irradiated cells; 266.7 ± 33.5 pg/mL (14.7 ± 2.6 pg/mL) for LEE-irradiated cells, p = 0.0969 (p = 0.0248) compared to non-irradiated cells, Kruskal-Wallis test; 226.6 ± 17.4 pg/mL (21.9 ± 8.1 pg/mL) for gamma-irradiated cells, p = 0.0070 (p = 0.0389) compared to non-irradiated cells, Kruskal-Wallis test] (**Figure 2D**).

### Irradiation Causes Decrease in CD56 Surface Expression

**Figure 3:**
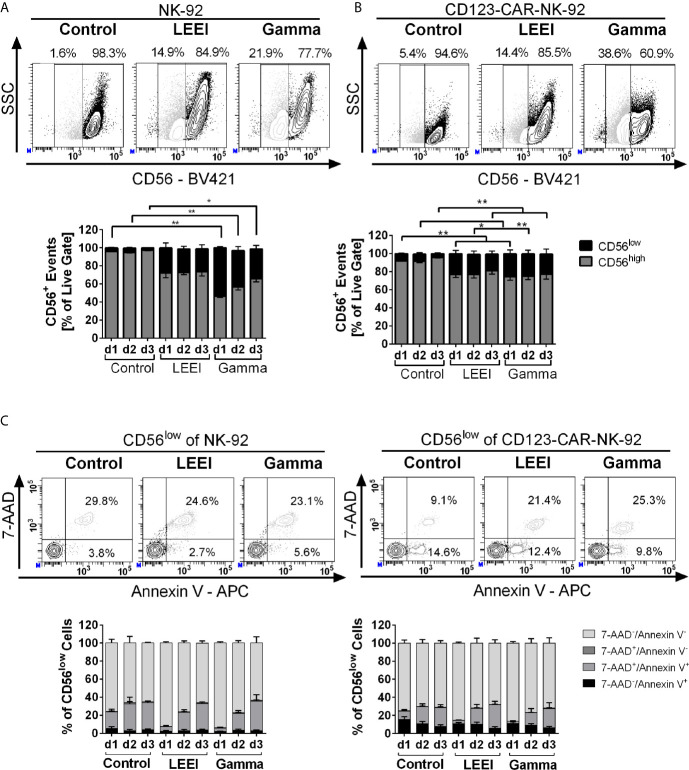
**Irradiation decreases surface expression of CD56. (A+B): NK-92 (**A**, n = 4) and CD123-CAR-NK-92 cells (**B**, n = 7) were stained with anti-human CD56 antibody. Surface expression levels of CD56 are indicated as CD56^low^ (black) and CD56^high^ (grey). Representative contour plots of non-irradiated (control), LEE-irradiated, and gamma-irradiated cells on day 3 post-irradiation are shown. **(C)** Analysis of the CD56^low^ subpopulation of NK-92 (left) and CD123-CAR-NK-92 cells (right) regarding apoptosis: 7-AAD^-^/Annexin V^-^ (lightest grey), 7-AAD^+^/Annexin V^-^ (dark grey), 7-AAD^+^/Annexin V^+^(light grey) and 7-AAD^−^/Annexin V^+^ (black). Representative contour plots of non-irradiated (control), LEE-irradiated, and gamma-irradiated cells on day 3 post-irradiation are shown. Values are indicated as means ± SEM, statistical significance is symbolized by asterisks (* for p ≤ 0.05 and ** for p ≤ 0.01, Kruskal-Wallis test adjusted for multiple comparisons by Dunn’s test).

In order to determine the effects of radiation on surface protein expression, flow cytometric analysis of CD56, a marker of mature functional NK-92 cells (33, 34), was performed (**Figure 3**). Overall surface expression of CD56 in NK-92 (**Figure 3A**) and CD123-directed CAR-NK-92 cells (**Figure 3B**) was not affected by gamma irradiation or LEEI, however, a population with considerable downregulation of CD56 (termed CD56^low^) of 20-50% emerged. To clarify, why the CD56 surface expression was downregulated after irradiation, the CD56^low^ (**Figure 3C**) and CD56^high^ (**Supplemental Figure S2B**) populations were analyzed regarding cell death distribution using Annexin V/7-AAD staining (re-analysis of data shown in **Figure 1D**). A schematic representation of the gating strategy is provided in **Supplemental Figure S2A**. The CD56^low^ subpopulation of NK-92 and CD123-CAR-NK-92 cells revealed a significantly higher proportion of Annexin V/7-AAD double positive cells (**Figure 3C**), compared to the CD56^high^ population (**Supplemental Figure S2B**) (p < 0.0001, Mann Whitney test). Still, only one third of the CD56^low^ population represented dead cells, whereas the majority of CD56^low^ cells were viable (negative for both 7-AAD and Annexin V).

Additional markers of NK cell activation (NKp46, NKG2D and NKp30) were analyzed and showed no differences in surface expression levels after irradiation in NK-92 and CD123-directed CAR-NK-92 cells (**Supplemental Figures S3A, B**).

In order to determine whether the CD56 downregulation of irradiated cells correlated to reduced cell metabolism, the metabolic activity of irradiated NK-92 and CD123-CAR-NK-92 cells was investigated by determining the bioreductive activity for generation of adenosine triphosphate (ATP), as well as cellular ATP levels (**Supplemental Figures S3C, D**). Non-irradiated as well as irradiated cells of both cell lines maintained their metabolic capacity up to 3 days post-treatment, except for a significant decrease in ATP levels of gamma-irradiated NK-92 cells on day 1 (63.4 ± 9.5%, p = 0.0257, compared to non-irradiated cells, Kruskal-Wallis test).

### Irradiation Causes DNA Damage and an Accumulation in G2/M Phase

**Figure 4:**
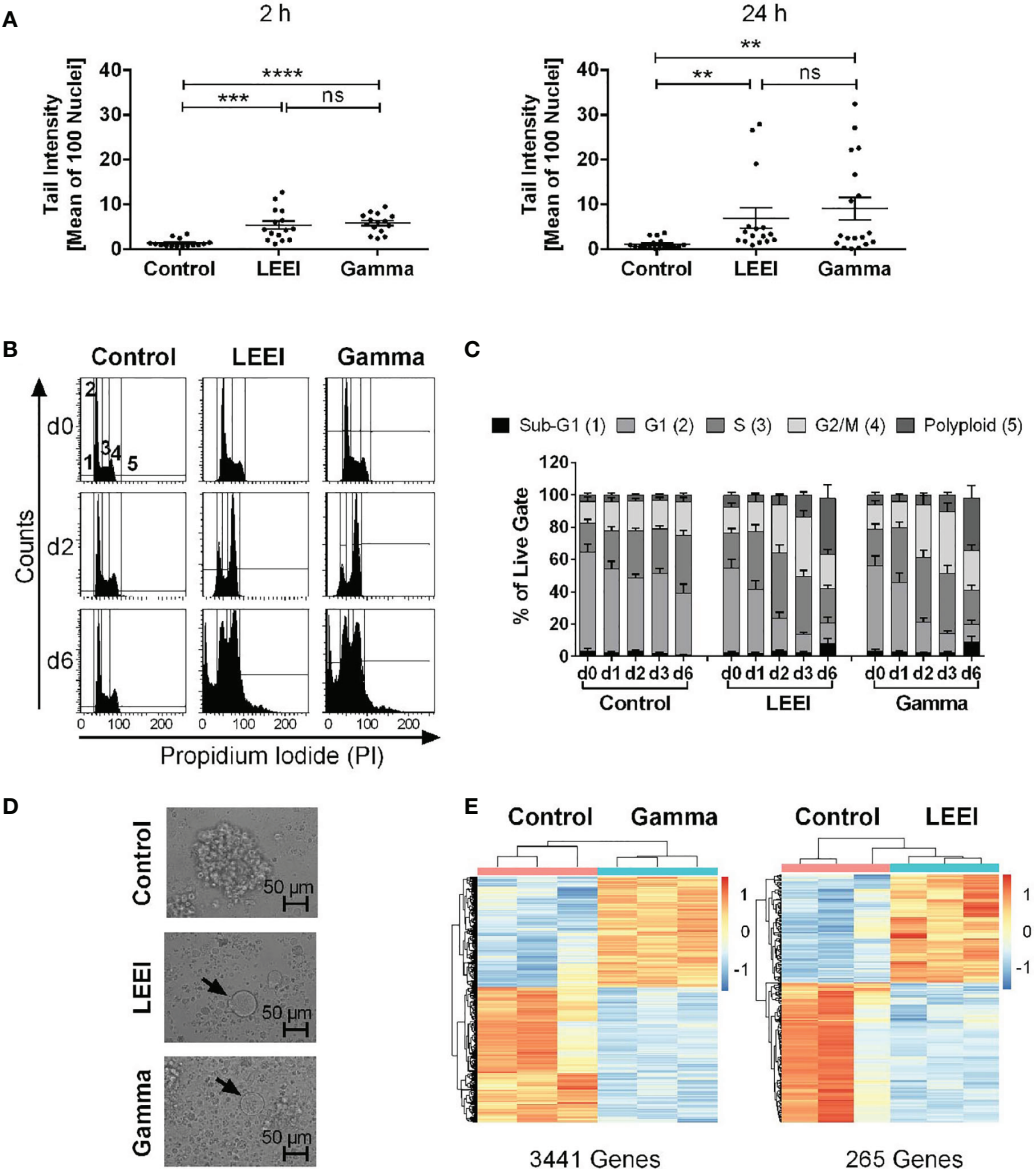
Gamma irradiation and LEEI result in DNA damage and an accumulation in G2/M phases of CD123-directed CAR-NK-92 cells. **(A)** Alkaline comet assays were performed with LEE- and gamma-irradiated versus non-irradiated CD123-directed CAR-NK-92 cells 2 h (left) or 24 h (right) after treatment to determine irradiation-induced DNA strand breaks. Values are depicted as means ± SEM (n = 6 independent experiments, each carried out in technical triplicates, minimum number of 100 cell nuclei analyzed per sample). Statistical significance is symbolized by asterisks (ns for p > 0.05, * for p ≤ 0.05, ** for p ≤ 0.01, *** for p ≤ 0.001, and **** for p ≤ 0.0001, Kruskal-Wallis test adjusted for multiple comparisons by Dunn’s test). **(B)** CD123-CAR-NK-92 cells (n = 8) were fixed and stained with propidium iodide (PI). Representative histograms of non-irradiated (control, left), LEE-irradiated (middle), and gamma-irradiated (right) cells are shown 2 h (d0, top), 2 days (d2, middle), and 6 days (d6, bottom) after treatment. Gating indicates cells defined as Sub-G1- (1), G1- (2), S- (3), G2/M phase (4) or polyploid (5). **(C)** Quantification of PI-staining of CD123-directed CAR-NK-92 (n = 8). Values are indicated as means ± SEM. **(D)** Light microscopy of non-irradiated (control, top), LEE-irradiated (center), and gamma-irradiated (bottom) CD123-directed CAR-NK-92 cells on day 10 post-irradiation. Arrows show enlarged cells. **(E)** Preliminary results of gene expression levels of gamma-irradiated (left) and LEE-irradiated (right) CD123-directed CAR-NK-92 cells both compared to non-irradiated cells 2 h after treatment. Heat maps of normalized, variance-stabilized and per gene standardized expression values of three technical replicates show up- (red) or down-regulated (blue) genes. The total number of significantly differentially regulated genes is indicated below (FDR < 0.01).

Since the induction of DNA damage is a key effect of radiation and necessary to guarantee proliferation inhibition, the alkaline comet assay was performed to investigate DNA strand break formation and a potential DNA repair response after gamma irradiation or LEEI. CD123-directed CAR-NK-92 cells were investigated 2 h (**Figure 4A**, left) and 24 h (**Figure 4A**, right) post-irradiation. Both gamma- and LEE-irradiated cells showed higher TIs than the untreated controls, thus indicating induction of DNA damage (non-irradiated control 2 h: 1.34 ± 0.23%; LEE-irradiated 2 h: 5.37 ± 0.91%, p = 0.0004; gamma-irradiated 2 h: 5.79 ± 0.56%, p < 0.0001; non-irradiated control 24 h: 1.10 ± 0.28%; LEE-irradiated 24 h: 6.94 ± 2.25%, p = 0.0017; gamma-irradiated 24 h: 9.04 ± 2.50%, p = 0.0094;Kruskall-Wallis test compared to non-irradiated cells). Comparison of the mean TI values 2 and 24 h after irradiation indicated no substantial DNA repair, but rather progressive DNA damage. TI values after LEEI and gamma irradiation were statistically not significantly different at either time point.

These data led to the hypothesis that accumulation of DNA damage and activation of DNA damage checkpoints cause irradiated cells to arrest in the G2/M phases (35). Therefore, the distribution of cell cycle phases was analyzed *via* PI staining and, indeed, revealed an increasing amount of cells in G2/M phases after irradiation (**Figures 4B, C**). The percentage of cells in the G1 phase decreased from day 0 after irradiation to day 3, whereas the percentage of cells in the G2/M phases increased from day 2 (up to 58%). Increased numbers of apoptotic cells in the Sub-G1 phase were detected after LEEI and gamma irradiation (up to 20% on day 6). Both irradiation methods also led to the generation of cells with an increased DNA content (up to 56% on day 6), indicating occurrence of polyploid cells. Morphologically, both LEE- and gamma-irradiated cells showed a drastic increase in cell size as well as dissolution of cell clusters accompanied by vast amounts of debris (**Figure 4D**).

### LEEI Results in Decreased Differential Gene Expression Compared to Gamma Irradiation

In order to analyze the impact of the irradiation-induced DNA damage on gene expression, NGS-based measurement of the transcriptome of CD123-directed CAR-NK-92 cells was performed 2 h after LEEI or gamma irradiation (**Figure 4E**). In these initial experiments, we found a striking difference in the number of differentially regulated genes between both irradiation types: 2 h post-irradiation, gamma-irradiated cells showed 3441 differentially regulated genes (FDR < 0.01), whereas LEE-irradiated cells showed only 265 differentially regulated genes (FDR < 0.01), when compared to non-irradiated control cells (**Figure 4E**). There were 193 common genes, which were differentially expressed after LEEI as well as after gamma irradiation.

Over-representation analysis (ORA) based on the Reactome database identified 35 pathways enriched in both LEEI or gamma irradiation *vs*. non-irradiated samples (**Supplemental Table S1**). These included pathways for non-sense mediated decay and pathways involved in translation. Genes differentially expressed in the comparison of gamma- and non-irradiated cells were additionally enriched in pathways related to nucleotide excision repair (NER), DNA damage and cell cycle regulation (**Supplemental Table S1**). The only pathway exclusively enriched in LEEI-treated cells (and not after gamma irradiation) was the RAF-independent MAPK1/3 activation pathway (**Supplemental Table S1**).

## Discussion

One important obstacle of (CAR-)NK-92 therapy is the safe inactivation of these therapeutic cells prior to application, which is crucial for the prevention of secondary tumor development (2, 36). Finding a balance between preventing the proliferation while maintaining a high cytotoxic potential is therefore of paramount importance. Especially on account of its “off-the-shelf” availability for allogeneic transfusion (2, 36), CAR-NK-92 therapy is currently used as an alternative to CAR-T cell therapy, and could also be successfully applied as a supplemental therapy.

Several publications have previously shown that gamma irradiation at a dose of 10 Gy is suitable for inactivation of (CAR-)NK-92 cells *in vitro* (37–44), in mouse models (38, 40, 43–46), as well as in first clinical trials (10, 11, 47). As an alternative method, we recently described the application of LEEI to inactivate NK-92 cells and demonstrated proof of concept that this technique can indeed render these cells proliferation-incompetent (18). Addressing the question whether it might be used as an alternative to gamma irradiation, we here provide a detailed comparison between both irradiation methods for the inactivation of NK-92 or CD123-directed CAR-NK-92 cells. Analysis of cell number and viability showed that both irradiation methods fully cease proliferation.

On a functional level, the cytotoxic potential of irradiated NK-92 and CD123-CAR-NK-92 cells was stably maintained for up to two days post-irradiation. Other reports in the literature confirm that *in vitro* cytotoxicity as well as cytokine secretion are maintained for 24 - 48 h after gamma irradiation (37, 38, 40, 41, 44). Three days after irradiation, we observed a reduction in cytotoxic activity, which was statistically significant only for gamma irradiation (the difference between gamma irradiation and LEEI was not statistically significant). This maintenance of cytotoxic activity could be due to the short exposure time of electron beams or due to the fact that LEEI initiates more direct damage to the DNA through targeted hits from electrons, while the effect of gamma radiation is predominantly mediated by secondary reactions from ROS, which result in increased oxidative stress. On day three post-irradiation, a decrease in the effector molecules sFasL and perforin was observed, whereas other soluble analytes, including IL-17A, granzyme B, granulysin, and IFNγ, were not influenced by irradiation or even showed a slight irradiation-induced increase. In accordance with this data, Nowakowska et al. described a decrease of sFasL levels, an increase of IFNγ levels as well as constant levels of granzyme B after gamma irradiation of Her2-directed CAR-NK-92 cells (37). Furthermore, irradiation-induced increases of IFNγ-levels were described in T cells (48). Interestingly, different cytokine secretion patterns were observed when comparing NK-92-mediated lysis of K562 cells and CAR-NK-92-mediated lysis of KG-1 cells, which could indicate that different combinations of effector molecules are generated in unmodified NK-92 cells and CAR-NK-92 cells upon interaction with target cells.

Irradiation had no impact on the total percentage of CD56^+^ (CAR-)NK-92 cells, as previously described in literature (18, 41), but flow cytometry showed an emerging CD56^low^ population in NK-92 and CD123-directed CAR-NK-92 cells after irradiation. As far as we are aware, this has never been described before. In our analyses, secretion levels of effector molecules and lysis of target cells were mainly maintained after irradiation, despite the negative regulation of CD56 surface expression. Therefore, we conclude that this CD56^low^ population is phenotypically and functionally different from the CD56^dim^ population of primary NK cells, which is characterized by a high cytotoxic potential, compared to CD56^bright^ NK cells (49, 50). Further examination of this CD56^low^ cell population by Annexin V and 7-AAD staining revealed that decreased CD56 surface expression could not be explained by increased cell death with subsequent downregulation of this marker. As the CD56 level is an indicator of NK cell activation and functional potential (51), the observed phenotype might indicate irradiation-induced exhaustion of the cells. However, improved analyses about this is hampered due to the lack of CD16 in NK-92, compared to primary human NK cells, where CD16 in combination with CD56 allows differentiation between cytotoxic and immunoregulatory NK cells. On the other hand, in our study, the irradiation-induced DNA damage had no impact on the surface expression of the activation receptors NKp46, NKG2D and NKp30, which is also in line with current literature (37, 41, 43).

Cell cycle analysis revealed an accumulation of irradiated cells in the G2/M phases and might reflect the activation of DNA damage checkpoints (35). First preliminary analyses of gene expression levels revealed a remarkable difference between both irradiation methods with gamma irradiation resulting in a more than 12-fold higher number of differentially regulated genes than LEEI. It might be assumed that the effects on DNA and gene expression levels are based on differences in the dose rate. The required number of targeted hits by LEEI is achieved within a very short time period (< 1 s), whereas the cells experience more secondary attacks over several minutes, which are required to reach 10 Gy with gamma irradiation. Jochems et al. previously analyzed gene expression of gamma-irradiated haNK cells (“high affinity”, genetically modified NK-92 cells) and found that the majority of differentially expressed genes was associated with cellular activation (52). In our study, both LEEI and gamma irradiation led to differentially expressed genes enriched in 35 different pathways when compared to non-irradiated cells. However, only one pathway could be identified which was enriched specifically after LEEI, the RAF-independent MAPK1/3 pathway. This enzyme cascade, that plays a central role in intracellular transmission of extracellular signals, is activated by several extracellular stimuli, including UV radiation (53, 54). In contrast, 286 pathways enriched for differentially regulated genes were found only after gamma irradiation. Included is the NER system. Given the observation that both LEEI and gamma irradiation led to similar DNA damage *via* strand breaks, this finding might indicate that lesions targeted by NER are more enriched upon gamma irradiation. Such lesions are mainly bulky photo-adducts or thymine dimers induced by UV light and by ROS (55). They are more likely to be secondary effects of radiation induced radicals in the cells, whereas the direct effects of ionizing radiation are DNA strand breaks. Therefore, although the effects on cellular viability are similar, both irradiation technologies seem to have different impacts on the overall cellular functions. A further in-depth analysis of these gene expression studies, which should involve the validation of candidate genes, might therefore reveal fundamental information on the effects of ionizing radiation on cells.

In summary, LEEI is a suitable alternative to state-of-the-art gamma irradiation as it yields comparably inactivated cellular products with a high cytotoxic activity and moreover provides many logistic advantages. Specifically, LEEI has low shielding requirements, which enables installation of radiation facilities in a standard laboratory surrounding, a very accurate and reproducible irradiation, and fast processing due to the high dose rate (18). Nevertheless, it has to be critically mentioned that the irradiation device used for LEEI is a research-scale prototype and still requires optimization for standardized clinical use. To fully leverage these advantages, the currently existing irradiation modules for LEEI have to be transformed into GMP-compatible devices, including an in-line process parameter control. Thereby, this technique could find applications in the automated processing of ATMPs for clinical trials.

The crucial next step will be to investigate and compare the effects of non-irradiated, LEE-irradiated, and gamma-irradiated CAR-NK-92 cells *in vivo*. Contrary to the project hypothesis, some studies described in literature have shown that non-irradiated cells can be applied safely without persisting or causing secondary tumors in mice (56, 57). This could evoke the impression that irradiation of NK-92 cells might be redundant, however, in this case the large differences between immunodeficient mouse models and the complex human organism have to be kept in mind. The fact that non-irradiated NK-92 cells do not persist in NSG mice might for instance be owed to the absence of cytokines. NK-92 are known to grow IL-2 dependent and show rapid cell death upon IL-2 withdrawal *in vitro* (58). For this reason, some *in vivo* models described in literature add IL-2 to the therapeutic regimen in order to enhance NK-92 persistence and efficacy (59–61). Alternatively, the cell line NK-92MI, which is genetically modified to produce IL-2 (39), can be used as a cytokine-independent effector cell population. Indeed, Liu et al. showed that non-irradiated CD19-directed CAR-NK-92MI cells persist and proliferate in NOD-SCID as well as in NSG mice (42). Therefore, it is still likely that non-irradiated (CAR-)NK-92 cells could proliferate in patients. Analyzing the persistence of non-irradiated and irradiated cells in mice after adding cytokines, or in humanized mice could provide interesting information in this context. Furthermore, in the future, the observed high *in vitro* functionality of LEE-irradiated cells should be confirmed by comparing the therapeutic effects of LEE- and gamma-irradiated cells *in vivo*.

## Data Availability Statement

The data presented in the study are online available and are deposited in the Gene Expression Omnibus (GEO) repository, accession number GSE166976. Online available under: https://www.ncbi.nlm.nih.gov/geo/query/acc.cgi?acc=GSE166976.

## Author Contributions

AB, CB, KR, GRM, SU, UK-B, UKöh, AD, and SF designed the research. LW, A-KK, CS, SB, DL, CB, and GRM performed the experiments. BS and MT designed and constructed the irradiation module. SK, AS, and MM designed and produced the CAR-NK-92 cells. LW, A-KK, CZ, SD, UT, SK, AS, MM, JB, UKön, BS, MT, GRM, SU, UK-B, AD, and SF analyzed and interpreted results. CK and KR analyzed the NGS data. LW, A-KK, CZ, SD, GRM, SU, UK-B, AD, and SF created figures and wrote the manuscript. UKöh reviewed the manuscript. All authors contributed to the article and approved the submitted version.

## Funding

This research was funded by the MAVO-grant “ELITE-NK cells” of the Fraunhofer society.

## Conflict of Interest

The authors declare that the research was conducted in the absence of any commercial or financial relationships that could be construed as a potential conflict of interest.

## References

[1] Suck G Odendahl M Nowakowska P Seidl C Wels WS Klingemann HG. Nk-92: An ‘Off-the-Shelf Therapeutic’ for Adoptive Natural Killer Cell-Based Cancer Immunotherapy. Cancer Immunol Immunother (2016) 65:485–92. 10.1007/s00262-015-1761-x PMC11029582 26559813

[2] Kloess S Kretschmer A Stahl L Fricke S Koehl U. Car-Expressing Natural Killer Cells for Cancer Retargeting. Transfus Med Hemother (2019) 46:4–13. 10.1159/000495771 31244577 PMC6558329

[3] Testa U Pelosi E Castelli G. CD123 as a Therapeutic Target in the Treatment of Hematological Malignancies. Cancers (Basel) (2019) 111358. 10.3390/cancers11091358 PMC6769702 31547472

[4] Arai N Homma M Abe M Baba Y Murai S Watanuki M. Impact of CD123 Expression, Analyzed by Immunohistochemistry, on Clinical Outcomes in Patients With Acute Myeloid Leukemia. Int J Hematol (2019) 109:539–44. 10.1007/s12185-019-02616-y 30847774

[5] Han L Jorgensen JL Brooks C Shi C Zhang Q Nogueras González GM. Antileukemia Efficacy and Mechanisms of Action of SL-101, a Novel Anti-Cd123 Antibody Conjugate, in Acute Myeloid Leukemia. Clin Cancer Res (2017) 23:3385–95. 10.1158/1078-0432.CCR-16-1904 PMC5496806 28096272

[6] Zahran AM Aly SS Rayan A El-Badawy O Fattah MA Ali AM. Survival Outcomes of CD34+CD38-LSCs and Their Expression of CD123 in Adult AML Patients. Oncotarget (2018) 9:34056–65. 10.18632/oncotarget.26118 PMC6183348 30344921

[7] Kloess S Oberschmidt O Dahlke J Vu X-K Neudoerfl C Kloos A. Preclinical Assessment of Suitable Natural Killer Cell Sources for Chimeric Antigen Receptor Natural Killer-Based “Off-the-Shelf” Acute Myeloid Leukemia Immunotherapies. Hum Gene Ther (2019) 30:381–401. 10.1089/hum.2018.247 30734584

[8] Rezvani K Rouce RH. The Application of Natural Killer Cell Immunotherapy for the Treatment of Cancer. Front Immunol (2015) 6:578. 10.3389/fimmu.2015.00578 26635792 PMC4648067

[9] Zhang C Oberoi P Oelsner S Waldmann A Lindner A Tonn T. Chimeric Antigen Receptor-Engineered NK-92 Cells: An Off-the-Shelf Cellular Therapeutic for Targeted Elimination of Cancer Cells and Induction of Protective Antitumor Immunity. Front Immunol (2017) 8:533. 10.3389/fimmu.2017.00533 28572802 PMC5435757

[10] Arai S Meagher R Swearingen M Myint H Rich E Martinson J. Infusion of the Allogeneic Cell Line NK-92 in Patients With Advanced Renal Cell Cancer or Melanoma: A Phase I Trial. Cytotherapy (2008) 10:625–32. 10.1080/14653240802301872 18836917

[11] Tonn T Schwabe D Klingemann HG Becker S Esser R Koehl U. Treatment of Patients With Advanced Cancer With the Natural Killer Cell Line NK-92. Cytotherapy (2013) 15:1563–70. 10.1016/j.jcyt.2013.06.017 24094496

[12] Yamamori T Yasui H Yamazumi M Wada Y Nakamura Y Nakamura H. Ionizing Radiation Induces Mitochondrial Reactive Oxygen Species Production Accompanied by Upregulation of Mitochondrial Electron Transport Chain Function and Mitochondrial Content Under Control of the Cell Cycle Checkpoint. Free Radic Biol Med (2012) 53:260–70. 10.1016/j.freeradbiomed.2012.04.033 22580337

[13] Smith TA Kirkpatrick DR Smith S Smith TK Pearson T Kailasam A. Radioprotective Agents to Prevent Cellular Damage Due to Ionizing Radiation. J Transl Med (2017) 15:232. 10.1186/s12967-017-1338-x 29121966 PMC5680756

[14] Maier P Hartmann L Wenz F Herskind C. Cellular Pathways in Response to Ionizing Radiation and Their Targetability for Tumor Radiosensitization. Int J Mol Sci (2016) 17:102. 10.3390/ijms17010102 PMC4730344 26784176

[15] Feng K Divers E Ma Y Li J. Inactivation of a Human Norovirus Surrogate, Human Norovirus Virus-Like Particles, and Vesicular Stomatitis Virus by Gamma Irradiation. Appl Environ Microbiol (2011) 77:3507–17. 10.1128/AEM.00081-11 PMC3126457 21441330

[16] Wu Q Allouch A Martins I Brenner C Modjtahedi N Deutsch E. Modulating Both Tumor Cell Death and Innate Immunity Is Essential for Improving Radiation Therapy Effectiveness. Front Immunol (2017) 8:613. 10.3389/fimmu.2017.00613 28603525 PMC5445662

[17] Wang J-s Wang H-j Qian H-l. Biological Effects of Radiation on Cancer Cells. Military Med Res (2018) 5:1–10. 10.1186/s40779-018-0167-4 PMC6026344 29958545

[18] Fertey J Thoma M Beckmann J Bayer L Finkensieper J Reißhauer S. Automated Application of Low Energy Electron Irradiation Enables Inactivation of Pathogen- and Cell-Containing Liquids in Biomedical Research and Production Facilities. Sci Rep (2020) 10:12786. 10.1038/s41598-020-69347-7 32732876 PMC7393095

[19] Rögner F-H Wetzel C Röder O Gotzmann G. Sterilization of Surgical Instruments Using Mini Electron Accelerators: In Proceedings of the 52nd Annual Technical Conference 2009. Santa Clara, CA, USA: Society of Vacuum Coaters. (2009).

[20] Gotzmann G Portillo J Wronski S Kohl Y Gorjup E Schuck H. Low-Energy Electron-Beam Treatment as Alternative for on-Site Sterilization of Highly Functionalized Medical Products – A Feasibility Study. Radiat Phys Chem (2018) 150:9–19. 10.1016/j.radphyschem.2018.04.008

[21] Fertey J Bayer L Grunwald T Pohl A Beckmann J Gotzmann G. Pathogens Inactivated by Low-Energy-Electron Irradiation Maintain Antigenic Properties and Induce Protective Immune Responses. Viruses (2016) 8:319. 10.3390/v8110319 PMC5127033 27886076

[22] Suerth JD Morgan MA Kloess S Heckl D Neudörfl C Falk CS. Efficient Generation of Gene-Modified Human Natural Killer Cells *Via* Alpharetroviral Vectors. J Mol Med (Berl) (2016) 94:83–93. 10.1007/s00109-015-1327-6 26300042

[23] Ziemann C Jackson P Brown R Attik G Rihn BH Creutzenberg O. Quartz-Containing Ceramic Dusts: *In vitro* Screening of the Cytotoxic, Genotoxic and Pro-inflammatory Potential of 5 Factory Samples. J. Phys. Conf. Ser (2009) 151:12022. 10.1088/1742-6596/151/1/012022

[24] Schubert M Lindgreen S Orlando L. AdapterRemoval v2: Rapid Adapter Trimming, Identification, and Read Merging. BMC Res Notes (2016) 9:88. 10.1186/s13104-016-1900-2 26868221 PMC4751634

[25] Kim D Paggi JM Park C Bennett C Salzberg SL. Graph-Based Genome Alignment and Genotyping With HISAT2 and HISAT-Genotype. Nat Biotechnol (2019) 37:907–15. 10.1038/s41587-019-0201-4 PMC7605509 31375807

[26] Frankish A Diekhans M Ferreira A-M Johnson R Jungreis I Loveland J. GENCODE Reference Annotation for the Human and Mouse Genomes. Nucleic Acids Res (2019) 47:D766–73. 10.1093/nar/gky955 PMC6323946 30357393

[27] Anders S Pyl PT Huber W. Htseq–a Python Framework to Work With High-Throughput Sequencing Data. Bioinformatics (2015) 31:166–9. 10.1093/bioinformatics/btu638 PMC4287950 25260700

[28] Kämpf C Specht M Scholz A Puppel S-H Doose G Reiche K. Uap: Reproducible and Robust HTS Data Analysis. BMC Bioinf (2019) 20:664. 10.1186/s12859-019-3219-1 PMC6909466 31830916

[29] Love MI Huber W Anders S. Moderated Estimation of Fold Change and Dispersion for RNA-seq Data With Deseq2. Genome Biol (2014) 15:550. 10.1186/s13059-014-0550-8 25516281 PMC4302049

[30] Jassal B Matthews L Viteri G Gong C Lorente P Fabregat A. The Reactome Pathway Knowledgebase. Nucleic Acids Res (2020) 48:D498–503. 10.1093/nar/gkz1031 PMC7145712 31691815

[31] Yu G He Q-Y. ReactomePA: An R/Bioconductor Package for Reactome Pathway Analysis and Visualization. Mol Biosyst (2016) 12:477–9. 10.1039/C5MB00663E 26661513

[32] Yu G Wang L-G Han Y He Q-Y. clusterProfiler: An R Package for Comparing Biological Themes Among Gene Clusters. OMICS (2012) 16:284–7. 10.1089/omi.2011.0118 PMC3339379 22455463

[33] van Acker HH Capsomidis A Smits EL van Tendeloo VF. CD56 in the Immune System: More Than a Marker for Cytotoxicity? Front Immunol (2017) 8:892. 10.3389/fimmu.2017.00892 28791027 PMC5522883

[34] Mace EM Gunesch JT Dixon A Orange JS. Human NK Cell Development Requires CD56-mediated Motility and Formation of the Developmental Synapse. Nat Commun (2016) 7:12171. 10.1038/ncomms12171 27435370 PMC4961740

[35] Stark GR Taylor WR. Analyzing the G2/M Checkpoint. In: Schönthal AH, editor. Checkpoint Controls and Cancer: Volume 1: Reviews and Model Systems. Totowa, NJ: Humana Press (2004). p. 51–82. 10.1385/1-59259-788-2:051 15187249

[36] Mehta RS Rezvani K. Chimeric Antigen Receptor Expressing Natural Killer Cells for the Immunotherapy of Cancer. Front Immunol (2018) 9:283. 10.3389/fimmu.2018.00283 29497427 PMC5818392

[37] Nowakowska P Romanski A Miller N Odendahl M Bonig H Zhang C. Clinical Grade Manufacturing of Genetically Modified, CAR-expressing Nk-92 Cells for the Treatment of ErbB2-positive Malignancies. Cancer Immunol Immunother (2018) 67:25–38. 10.1007/s00262-017-2055-2 28879551 PMC11028154

[38] Schönfeld K Sahm C Zhang C Naundorf S Brendel C Odendahl M. Selective Inhibition of Tumor Growth by Clonal NK Cells Expressing an ErbB2/HER2-specific Chimeric Antigen Receptor. Mol Ther (2015) 23:330–8. 10.1038/mt.2014.219 PMC4445620 25373520

[39] Tam YK Maki G Miyagawa B Hennemann B Tonn T Klingemann HG. Characterization of Genetically Altered, Interleukin 2-Independent Natural Killer Cell Lines Suitable for Adoptive Cellular Immunotherapy. Hum Gene Ther (1999) 10:1359–73. 10.1089/10430349950018030 10365666

[40] Zhang C Burger MC Jennewein L Genßler S Schönfeld K Zeiner P. Erbb2/HER2-Specific NK Cells for Targeted Therapy of Glioblastoma. J Natl Cancer Inst (2016) 108:djv375. 10.1093/jnci/djv375 26640245

[41] Jochems C Hodge JW Fantini M Fujii R Morillon YM Greiner JW. An NK Cell Line (haNK) Expressing High Levels of Granzyme and Engineered to Express the High Affinity CD16 Allele. Oncotarget (2016) 7:86359–73. 10.18632/oncotarget.13411 PMC5341330 27861156

[42] Liu Q Xu Y Mou J Tang K Fu X Li Y. Irradiated Chimeric Antigen Receptor Engineered NK-92MI Cells Show Effective Cytotoxicity Against CD19+ Malignancy in a Mouse Model. Cytotherapy (2020) 22:552–62. 10.1016/j.jcyt.2020.06.003 32747298

[43] Montagner IM Penna A Fracasso G Carpanese D Dalla Pietà A Barbieri V. Anti-PSMA CAR-engineered Nk-92 Cells: An Off-the-shelf Cell Therapy for Prostate Cancer. Cells (2020) 9:1382. 10.3390/cells9061382 PMC7349573 32498368

[44] Jiang H Zhang W Shang P Zhang H Fu W Ye F. Transfection of Chimeric anti-CD138 Gene Enhances Natural Killer Cell Activation and Killing of Multiple Myeloma Cells. Mol Oncol (2014) 8:297–310. 10.1016/j.molonc.2013.12.001 24388357 PMC5528539

[45] Williams BA Wang X-H Leyton JV Maghera S Deif B Reilly RM. Cd16+Nk-92 and anti-CD123 Monoclonal Antibody Prolongs Survival in Primary Human Acute Myeloid Leukemia Xenografted Mice. Haematologica (2018) 103:1720–9. 10.3324/haematol.2017.187385 PMC6165813 29976748

[46] Tam YK Miyagawa B Ho VC Klingemann HG. Immunotherapy of Malignant Melanoma in a SCID Mouse Model Using the Highly Cytotoxic Natural Killer Cell Line NK-92. J Hematother (1999) 8:281–90. 10.1089/106161299320316 10417052

[47] Williams BA Law AD Routy B denHollander N Gupta V Wang X-H. A Phase I Trial of NK-92 Cells for Refractory Hematological Malignancies Relapsing After Autologous Hematopoietic Cell Transplantation Shows Safety and Evidence of Efficacy. Oncotarget (2017) 8:89256–68. 10.18632/oncotarget.19204 PMC5687687 29179517

[48] Gerber SA Sedlacek AL Cron KR Murphy SP Frelinger JG Lord EM. Ifn-γ Mediates the Antitumor Effects of Radiation Therapy in a Murine Colon Tumor. Am J Pathol (2013) 182:2345–54. 10.1016/j.ajpath.2013.02.041 PMC3668027 23583648

[49] Koehl U Brehm C Huenecke S Zimmermann S-Y Kloess S Bremm M. Clinical Grade Purification and Expansion of NK Cell Products for an Optimized Manufacturing Protocol. Front Oncol (2013) 3:118. 10.3389/fonc.2013.00118 23730623 PMC3656406

[50] Cooper MA Fehniger TA Caligiuri MA. The Biology of Human Natural Killer-Cell Subsets. Trends Immunol (2001) 22:633–40. 10.1016/s1471-4906(01)02060-9 11698225

[51] . Poznanski SM Ashkar AA. Shining Light on the Significance of NK Cell CD56 Brightness. Cell Mol Immunol (2018) 15:1071–3. 10.1038/s41423-018-0163-3 PMC6269508 30275534

[52] Jochems C Hodge JW Fantini M Tsang KY Vandeveer AJ Gulley JL. ADCC Employing an NK Cell Line (haNK) Expressing the High Affinity CD16 Allele With Avelumab, an anti-PD-L1 Antibody. Int J Cancer (2017) 141:583–93. 10.1002/ijc.30767 PMC5604238 28477372

[53] Rubinfeld H Seger R. The ERK Cascade as a Prototype of MAPK Signaling Pathways. Methods Mol Biol (2004) 250:1–28. 10.1385/1-59259-671-1:1 14755077

[54] Kim T Lawson MA. Gnrh Regulates Gonadotropin Gene Expression Through Nadph/Dual Oxidase-Derived Reactive Oxygen Species. Endocrinology (2015) 156:2185–99. 10.1210/en.2014-1709 PMC4430611 25849727

[55] Lee T-H Kang T-H. Dna Oxidation and Excision Repair Pathways. Int J Mol Sci (2019) 20:6092. 10.3390/ijms20236092 PMC6929053 31816862

[56] Chu J Deng Y Benson DM He S Hughes T Zhang J. CS1-Specific Chimeric Antigen Receptor (CAR)-Engineered Natural Killer Cells Enhance In Vitro and In Vivo Antitumor Activity Against Human Multiple Myeloma. Leukemia (2014) 28:917–27. 10.1038/leu.2013.279 PMC3967004 24067492

[57] Han J Chu J Keung Chan W Zhang J Wang Y Cohen JB. Car-Engineered NK Cells Targeting Wild-Type EGFR and EGFRvIII Enhance Killing of Glioblastoma and Patient-Derived Glioblastoma Stem Cells. Sci Rep (2015) 5:11483. 10.1038/srep11483 26155832 PMC4496728

[58] Gong JH Maki G Klingemann HG. Characterization of a Human Cell Line (NK-92) With Phenotypical and Functional Characteristics of Activated Natural Killer Cells. Leukemia (1994) 8:652–8. 8152260

[59] Zhang Q Tian K Xu J Zhang H Li L Fu Q. Synergistic Effects of Cabozantinib and EGFR-Specific Car-Nk-92 Cells in Renal Cell Carcinoma. J Immunol Res (2017) 2017:6915912. 10.1155/2017/6915912 29423418 PMC5750507

[60] Zhang Q Zhang H Ding J Liu H Li H Li H. Combination Therapy With EpCAM-CAR-NK-92 Cells and Regorafenib Against Human Colorectal Cancer Models. J Immunol Res (2018) 2018:4263520. 10.1155/2018/4263520 30410941 PMC6205314

[61] Seidel D Shibina A Siebert N Wels WS Reynolds CP Huebener N. Disialoganglioside-Specific Human Natural Killer Cells are Effective Against Drug-Resistant Neuroblastoma. Cancer Immunol Immunother (2015) 64:621–34. 10.1007/s00262-015-1669-5 PMC11029162 25711293

