# Supplementary material for: Low Energy Electron Irradiation Is a Potent Alternative to Gamma Irradiation for the Inactivation of (CAR-)NK-92 Cells in ATMP Manufacturing
Source: Front Immunol. 2021 Jun 4;12:684052. doi: 10.3389/fimmu.2021.684052 (PMC8212864; doi:10.3389/fimmu.2021.684052)
Supplement: Supplementary file 1 [file DataSheet_1.docx]

Supplementary Material

**Supplementary Figure 1.** Representative contour plots of flow cytometric analysis of NK-92 (left) and CD123-directed CAR-NK-92 (right) cells after 7-AAD and Annexin V staining.

**Supplementary Figure 2.** **(A)** Schematic gating strategy for flow cytometry analysis of 7-AAD and Annexin V staining of CD56^low^ and CD56^high^ subpopulation of NK-92 and CD123-CAR-NK-92 cells after irradiation. **(B)** After irradiation, CD56^high^ phenotype of NK-92 (left) and CD123-CAR-NK-92 cells (right) was analyzed regarding apoptosis: 7-AAD^-^/Annexin V^-^ (lightest grey), 7-AAD^+^/Annexin V^-^ (dark grey), 7-AAD^+^/Annexin V^+^ (light grey) and 7-AAD^-^/Annexin V^-^(black). Non-irradiated cells were used as a control. Representative contour plots of non-irradiated (control), LEE-irradiated, and gamma-irradiated cells of day 3 post-irradiation are shown. Data shown as means ± SEM.

**Supplementary Figure 3. (A+B)** NK-92 (**A**, n = 2) and CD123-CAR-NK-92 cells (**B**, n = 2) were stained with anti-human NKG2D, anti-human NKp46 and anti-human NKp30 antibodies after LEEI (grey) and gamma irradiation (white). Non-irradiated cells (black) were used as a control.
**(C+D)** Metabolic activity of LEE- (grey) and gamma- (white) irradiated NK-92 (**C**, n = 5) and CD123-CAR-NK-92 (**D**, n = 8) cells was measured on the basis of their bioreductive activity (top) or their ATP levels (bottom). Non-irradiated cells (black) were used as a control. Collected data were compared to the data from these cells on day one (100%). Data shown as mean*s* ± SEM, statistical significance is symbolized by asterisks (* for p ≤ 0.05, and ns for p > 0.05, Kruskal-Wallis test adjusted for multiple comparisons by Dunn’s test).

**Supplementary Table 1**: Enriched pathways identified by over-representation analysis.

| **Pathways enriched in … [vs. Non-irradiated]** | | |
| --- | --- | --- |
| **Both LEE- and Gamma-irradiated** | **Only LEE-irradiated** | **Only Gamma-irradiated** |
| Activation of the mRNA upon binding of the cap-binding complex and eIFs, and subsequent binding to 43S | RAF-independent MAPK1/3 activation | ABC transporter disorders |
| Cap-dependent Translation Initiation |  | ABC-family proteins mediated transport |
| Cristae formation |  | Activation of APC/C and APC/C:Cdc20 mediated degradation of mitotic proteins |
| Eukaryotic Translation Elongation |  | Activation of NF-kappaB in B cells |
| Eukaryotic Translation Initiation |  | Activation of the pre-replicative complex |
| Eukaryotic Translation Termination |  | Aggrephagy |
| Formation of a pool of free 40S subunits |  | Amino acids regulate mTORC1 |
| Formation of ATP by chemiosmotic coupling |  | Amplification of signal from unattached kinetochores via a MAD2 inhibitory signal |
| Formation of the ternary complex, and subsequently, the 43S complex |  | Amplification of signal from the kinetochores |
| GTP hydrolysis and joining of the 60S ribosomal subunit |  | Antigen processing: Ubiquitination & Proteasome degradation |
| Influenza Infection |  | Antigen processing-Cross presentation |
| Influenza Viral RNA Transcription and Replication |  | Antiviral mechanism by IFN-stimulated genes |
| L13a-mediated translational silencing of Ceruloplasmin expression |  | APC/C:Cdc20 mediated degradation of Cyclin B |
| Major pathway of rRNA processing in the nucleolus and cytosol |  | APC/C:Cdc20 mediated degradation of mitotic proteins |
| Metabolism of amino acids and derivatives |  | APC/C:Cdc20 mediated degradation of Securin |
| Nonsense Mediated Decay (NMD) enhanced by the Exon Junction Complex (EJC) |  | APC/C:Cdh1 mediated degradation of Cdc20 and other APC/C:Cdh1 targeted proteins in late mitosis/early G1 |
| Nonsense Mediated Decay (NMD) independent of the Exon Junction Complex (EJC) |  | APC/C-mediated degradation of cell cycle proteins |
| Nonsense-Mediated Decay (NMD) |  | APC:Cdc20 mediated degradation of cell cycle proteins prior to satisfation of the cell cycle checkpoint |
| Peptide chain elongation |  | APC-Cdc20 mediated degradation of Nek2A |
| Regulation of expression of SLITs and ROBOs |  | Apoptosis |
| Respiratory electron transport |  | Asparagine N-linked glycosylation |
| Respiratory electron transport, ATP synthesis by chemiosmotic coupling, and heat production by uncoupling proteins. |  | Assembly of the pre-replicative complex |
| Response of EIF2AK4 (GCN2) to amino acid deficiency |  | Asymmetric localization of PCP proteins |
| Ribosomal scanning and start codon recognition |  | AUF1 (hnRNP D0) binds and destabilizes mRNA |
| rRNA processing |  | Autodegradation of Cdh1 by Cdh1:APC/C |
| rRNA processing in the nucleus and cytosol |  | Autodegradation of the E3 ubiquitin ligase COP1 |
| Selenoamino acid metabolism |  | Autophagy |
| Selenocysteine synthesis |  | Beta-catenin independent WNT signaling |
| Signaling by Interleukins |  | Budding and maturation of HIV virion |
| Signaling by ROBO receptors |  | Calnexin/calreticulin cycle |
| SRP-dependent cotranslational protein targeting to membrane |  | Cdc20:Phospho-APC/C mediated degradation of Cyclin A |
| The citric acid (TCA) cycle and respiratory electron transport |  | CDK-mediated phosphorylation and removal of Cdc6 |
| Translation |  | CDT1 association with the CDC6:ORC:origin complex |
| Translation initiation complex formation |  | Cell Cycle Checkpoints |
| Viral mRNA Translation |  | Cellular response to heat stress |
|  |  | Cellular response to hypoxia |
|  |  | Chromatin modifying enzymes |
|  |  | Chromatin organization |
|  |  | Circadian Clock |
|  |  | Class I MHC mediated antigen processing & presentation |
|  |  | CLEC7A (Dectin-1) signaling |
|  |  | Complex I biogenesis |
|  |  | Constitutive Signaling by NOTCH1 HD+PEST Domain Mutants |
|  |  | Constitutive Signaling by NOTCH1 PEST Domain Mutants |
|  |  | Cooperation of Prefoldin and TriC/CCT in actin and tubulin folding |
|  |  | COPI-independent Golgi-to-ER retrograde traffic |
|  |  | Cross-presentation of soluble exogenous antigens (endosomes) |
|  |  | C-type lectin receptors (CLRs) |
|  |  | Cyclin A:Cdk2-associated events at S phase entry |
|  |  | Cyclin E associated events during G1/S transition |
|  |  | Cytosolic sensors of pathogen-associated DNA |
|  |  | Cytosolic tRNA aminoacylation |
|  |  | Deactivation of the beta-catenin transactivating complex |
|  |  | Deadenylation-dependent mRNA decay |
|  |  | Dectin-1 mediated noncanonical NF-kB signaling |
|  |  | Defective CFTR causes cystic fibrosis |
|  |  | Degradation of AXIN |
|  |  | Degradation of beta-catenin by the destruction complex |
|  |  | Degradation of DVL |
|  |  | Degradation of GLI1 by the proteasome |
|  |  | Degradation of GLI2 by the proteasome |
|  |  | Deubiquitination |
|  |  | Diseases of signal transduction by growth factor receptors and second messengers |
|  |  | Disorders of Developmental Biology |
|  |  | Disorders of Nervous System Development |
|  |  | Disorders of transmembrane transporters |
|  |  | DNA Damage Bypass |
|  |  | DNA Damage Recognition in GG-NER |
|  |  | DNA Repair |
|  |  | DNA Replication |
|  |  | DNA Replication Pre-Initiation |
|  |  | Downregulation of SMAD2/3:SMAD4 transcriptional activity |
|  |  | Downregulation of TGF-beta receptor signaling |
|  |  | Downstream signaling events of B Cell Receptor (BCR) |
|  |  | Downstream TCR signaling |
|  |  | Dual Incision in GG-NER |
|  |  | Dual incision in TC-NER |
|  |  | EML4 and NUDC in mitotic spindle formation |
|  |  | ER Quality Control Compartment (ERQC) |
|  |  | ER-Phagosome pathway |
|  |  | Export of Viral Ribonucleoproteins from Nucleus |
|  |  | FBXL7 down-regulates AURKA during mitotic entry and in early mitosis |
|  |  | Fc epsilon receptor (FCERI) signaling |
|  |  | FCERI mediated NF-kB activation |
|  |  | Folding of actin by CCT/TriC |
|  |  | Formation of HIV elongation complex in the absence of HIV Tat |
|  |  | Formation of HIV-1 elongation complex containing HIV-1 Tat |
|  |  | Formation of Incision Complex in GG-NER |
|  |  | Formation of RNA Pol II elongation complex |
|  |  | Formation of TC-NER Pre-Incision Complex |
|  |  | Formation of tubulin folding intermediates by CCT/TriC |
|  |  | G1/S DNA Damage Checkpoints |
|  |  | G1/S Transition |
|  |  | G2/M Checkpoints |
|  |  | G2/M Transition |
|  |  | Gap-filling DNA repair synthesis and ligation in GG-NER |
|  |  | Gap-filling DNA repair synthesis and ligation in TC-NER |
|  |  | Gene and protein expression by JAK-STAT signaling after Interleukin-12 stimulation |
|  |  | GLI3 is processed to GLI3R by the proteasome |
|  |  | Global Genome Nucleotide Excision Repair (GG-NER) |
|  |  | HDR through Homologous Recombination (HRR) |
|  |  | Hedgehog ligand biogenesis |
|  |  | Hedgehog 'off' state |
|  |  | Hedgehog 'on' state |
|  |  | Hh mutants abrogate ligand secretion |
|  |  | Hh mutants that don't undergo autocatalytic processing are degraded by ERAD |
|  |  | HIV elongation arrest and recovery |
|  |  | HIV Infection |
|  |  | HIV Life Cycle |
|  |  | HIV Transcription Elongation |
|  |  | HIV Transcription Initiation |
|  |  | Host Interactions of HIV factors |
|  |  | HSF1 activation |
|  |  | HSP90 chaperone cycle for steroid hormone receptors (SHR) |
|  |  | Inactivation of APC/C via direct inhibition of the APC/C complex |
|  |  | Inhibition of the proteolytic activity of APC/C required for the onset of anaphase by mitotic spindle checkpoint components |
|  |  | Initiation of Nuclear Envelope (NE) Reformation |
|  |  | Interactions of Rev with host cellular proteins |
|  |  | Interleukin-1 family signaling |
|  |  | Interleukin-1 signaling |
|  |  | Interleukin-12 family signaling |
|  |  | Interleukin-12 signaling |
|  |  | Intracellular signaling by second messengers |
|  |  | Intra-Golgi and retrograde Golgi-to-ER traffic |
|  |  | Intra-Golgi traffic |
|  |  | ISG15 antiviral mechanism |
|  |  | Late Phase of HIV Life Cycle |
|  |  | Loss of function of MECP2 in Rett syndrome |
|  |  | M Phase |
|  |  | Macroautophagy |
|  |  | MAPK family signaling cascades |
|  |  | MAPK6/MAPK4 signaling |
|  |  | Metabolism of non-coding RNA |
|  |  | Metabolism of polyamines |
|  |  | MicroRNA (miRNA) biogenesis |
|  |  | Mitochondrial biogenesis |
|  |  | Mitochondrial calcium ion transport |
|  |  | Mitochondrial protein import |
|  |  | Mitochondrial translation |
|  |  | Mitochondrial translation elongation |
|  |  | Mitochondrial translation initiation |
|  |  | Mitochondrial translation termination |
|  |  | Mitophagy |
|  |  | Mitotic Anaphase |
|  |  | Mitotic G1 phase and G1/S transition |
|  |  | Mitotic G2-G2/M phases |
|  |  | Mitotic Metaphase and Anaphase |
|  |  | Mitotic Prometaphase |
|  |  | Mitotic Spindle Checkpoint |
|  |  | mRNA Splicing |
|  |  | mRNA Splicing - Major Pathway |
|  |  | mRNA Splicing - Minor Pathway |
|  |  | Neddylation |
|  |  | Negative regulation of NOTCH4 signaling |
|  |  | NEP/NS2 Interacts with the Cellular Export Machinery |
|  |  | N-glycan trimming in the ER and Calnexin/Calreticulin cycle |
|  |  | NIK-->noncanonical NF-kB signaling |
|  |  | NOD1/2 Signaling Pathway |
|  |  | NOTCH1 Intracellular Domain Regulates Transcription |
|  |  | Notch-HLH transcription pathway |
|  |  | Nuclear Envelope (NE) Reassembly |
|  |  | Nuclear Envelope Breakdown |
|  |  | Nuclear import of Rev protein |
|  |  | Nucleotide Excision Repair |
|  |  | Nucleotide-binding domain, leucine rich repeat containing receptor (NLR) signaling pathways |
|  |  | Orc1 removal from chromatin |
|  |  | Organelle biogenesis and maintenance |
|  |  | Oxygen-dependent proline hydroxylation of Hypoxia-inducible Factor Alpha |
|  |  | p53-Dependent G1 DNA Damage Response |
|  |  | p53-Dependent G1/S DNA damage checkpoint |
|  |  | p53-Independent DNA Damage Response |
|  |  | p53-Independent G1/S DNA damage checkpoint |
|  |  | Pausing and recovery of HIV elongation |
|  |  | Pausing and recovery of Tat-mediated HIV elongation |
|  |  | PCP/CE pathway |
|  |  | Pervasive developmental disorders |
|  |  | Phosphorylation of the APC/C |
|  |  | PINK1-PRKN Mediated Mitophagy |
|  |  | PIP3 activates AKT signaling |
|  |  | Prefoldin mediated transfer of substrate to CCT/TriC |
|  |  | Processing of Capped Intron-Containing Pre-mRNA |
|  |  | Processing of SMDT1 |
|  |  | Programmed Cell Death |
|  |  | Protein localization |
|  |  | PTEN Regulation |
|  |  | Recognition of DNA damage by PCNA-containing replication complex |
|  |  | Regulation of activated PAK-2p34 by proteasome mediated degradation |
|  |  | Regulation of APC/C activators between G1/S and early anaphase |
|  |  | Regulation of Apoptosis |
|  |  | Regulation of HSF1-mediated heat shock response |
|  |  | Regulation of MECP2 expression and activity |
|  |  | Regulation of mitotic cell cycle |
|  |  | Regulation of mRNA stability by proteins that bind AU-rich elements |
|  |  | Regulation of ornithine decarboxylase (ODC) |
|  |  | Regulation of PLK1 Activity at G2/M Transition |
|  |  | Regulation of PTEN stability and activity |
|  |  | Regulation of RAS by GAPs |
|  |  | Regulation of RUNX2 expression and activity |
|  |  | Regulation of RUNX3 expression and activity |
|  |  | Regulation of TP53 Activity |
|  |  | Resolution of Sister Chromatid Cohesion |
|  |  | Response of Mtb to phagocytosis |
|  |  | Rev-mediated nuclear export of HIV RNA |
|  |  | RHO GTPase Effectors |
|  |  | RHO GTPases Activate Formins |
|  |  | RNA Polymerase II HIV Promoter Escape |
|  |  | RNA Polymerase II Pre-transcription Events |
|  |  | RNA Polymerase II Promoter Escape |
|  |  | RNA polymerase II transcribes snRNA genes |
|  |  | RNA Polymerase II Transcription Elongation |
|  |  | RNA Polymerase II Transcription Initiation |
|  |  | RNA Polymerase II Transcription Initiation And Promoter Clearance |
|  |  | RNA Polymerase II Transcription Pre-Initiation And Promoter Opening |
|  |  | RNA Polymerase II Transcription Termination |
|  |  | RNA Polymerase III Abortive And Retractive Initiation |
|  |  | RNA Polymerase III Transcription |
|  |  | RNA Polymerase III Transcription Initiation |
|  |  | RNA Polymerase III Transcription Initiation From Type 3 Promoter |
|  |  | RNA Polymerase III Transcription Termination |
|  |  | rRNA modification in the nucleus and cytosol |
|  |  | RUNX1 regulates transcription of genes involved in differentiation of HSCs |
|  |  | S Phase |
|  |  | SARS-CoV Infections |
|  |  | SCF(Skp2)-mediated degradation of p27/p21 |
|  |  | SCF-beta-TrCP mediated degradation of Emi1 |
|  |  | Selective autophagy |
|  |  | Separation of Sister Chromatids |
|  |  | Signaling by Hedgehog |
|  |  | Signaling by NOTCH |
|  |  | Signaling by NOTCH1 HD+PEST Domain Mutants in Cancer |
|  |  | Signaling by NOTCH1 in Cancer |
|  |  | Signaling by NOTCH1 PEST Domain Mutants in Cancer |
|  |  | Signaling by NOTCH2 |
|  |  | Signaling by NOTCH4 |
|  |  | Signaling by TGFB family members |
|  |  | Signaling by TGF-beta Receptor Complex |
|  |  | Signaling by the B Cell Receptor (BCR) |
|  |  | Signaling by WNT |
|  |  | SLBP Dependent Processing of Replication-Dependent Histone Pre-mRNAs |
|  |  | SLBP independent Processing of Histone Pre-mRNAs |
|  |  | SMAD2/SMAD3:SMAD4 heterotrimer regulates transcription |
|  |  | snRNP Assembly |
|  |  | Stabilization of p53 |
|  |  | SUMO E3 ligases SUMOylate target proteins |
|  |  | SUMOylation |
|  |  | SUMOylation of DNA damage response and repair proteins |
|  |  | SUMOylation of DNA replication proteins |
|  |  | SUMOylation of RNA binding proteins |
|  |  | Switching of origins to a post-replicative state |
|  |  | Synthesis of active ubiquitin: roles of E1 and E2 enzymes |
|  |  | Synthesis of DNA |
|  |  | Tat-mediated elongation of the HIV-1 transcript |
|  |  | Tat-mediated HIV elongation arrest and recovery |
|  |  | TCF dependent signaling in response to WNT |
|  |  | TCR signaling |
|  |  | Termination of translesion DNA synthesis |
|  |  | TGF-beta receptor signaling activates SMADs |
|  |  | The role of GTSE1 in G2/M progression after G2 checkpoint |
|  |  | TNFR2 non-canonical NF-kB pathway |
|  |  | TP53 Regulates Metabolic Genes |
|  |  | TP53 Regulates Transcription of DNA Repair Genes |
|  |  | Transcription of the HIV genome |
|  |  | Transcriptional activity of SMAD2/SMAD3:SMAD4 heterotrimer |
|  |  | Transcriptional regulation by RUNX1 |
|  |  | Transcriptional regulation by RUNX2 |
|  |  | Transcriptional regulation by RUNX3 |
|  |  | Transcriptional Regulation by TP53 |
|  |  | Transcription-Coupled Nucleotide Excision Repair (TC-NER) |
|  |  | Translesion Synthesis by POLH |
|  |  | Translesion synthesis by POLI |
|  |  | Translesion synthesis by POLK |
|  |  | Translesion synthesis by REV1 |
|  |  | Translesion synthesis by Y family DNA polymerases bypasses lesions on DNA template |
|  |  | Transport of Mature mRNA derived from an Intron-Containing Transcript |
|  |  | Transport of Mature Transcript to Cytoplasm |
|  |  | Transport of the SLBP Dependant Mature mRNA |
|  |  | tRNA Aminoacylation |
|  |  | tRNA processing |
|  |  | tRNA processing in the nucleus |
|  |  | Ubiquitin Mediated Degradation of Phosphorylated Cdc25A |
|  |  | Ubiquitin-dependent degradation of Cyclin D |
|  |  | Ub-specific processing proteases |
|  |  | UCH proteinases |
|  |  | Vif-mediated degradation of APOBEC3G |
|  |  | Viral Messenger RNA Synthesis |
|  |  | Vpu mediated degradation of CD4 |
